# Effect of Postbiotics Derived from *Lactobacillus rhamnosus* PB01 (DSM 14870) on Sperm Quality: A Prospective In Vitro Study

**DOI:** 10.3390/nu16111781

**Published:** 2024-06-06

**Authors:** Sihan Liu, Hiva Alipour, Vladimir Zachar, Ulrik Schiøler Kesmodel, Fereshteh Dardmeh

**Affiliations:** 1 Regenerative Medicine Group, Department of Health Science and Technology, Aalborg University, 9260 Gistrup, Denmark; hiva@hst.aau.dk (H.A.); vlaz@hst.aau.dk (V.Z.); 2 Department of Clinical Medicine, Aalborg University, 9260 Gistrup, Denmark; uske@dcm.aau.dk; 3 Department of Obstetrics and Gynecology, Aalborg University Hospital, 9000 Aalborg, Denmark

**Keywords:** probiotics, postbiotics, sperm motility, DNA fragmentation, *Lactobacillus rhamnosus*

## Abstract

Vaginally administered postbiotics derived from *Lactobacillus* were recently demonstrated to be effective in alleviating bacterial vaginosis and increasing pregnancy rates. However, their potential effect on sperm quality has not been well investigated. This controlled in vitro study aimed to assess the dose- and time-dependent effects of postbiotics derived from *Lactobacillus rhamnosus* PB01 (DSM 14870) on sperm quality parameters. The experiment was conducted in vitro to eliminate potential confounding factors from the female reproductive tract and vaginal microbiota. Sperm samples from 18 healthy donors were subjected to analysis using Computer-Aided Sperm Analysis (CASA) in various concentrations of postbiotics and control mediums at baseline, 60 min, and 90 min of incubation. Results indicated that lower postbiotic concentration (PB5) did not adversely affect sperm motility, kinematic parameters, sperm DNA fragmentation, and normal morphology at any time. However, concentrations exceeding 15% demonstrated a reduction in progressively motile sperm and a negative correlation with non-progressively motile sperm at all time points. These findings underscore the importance of balancing postbiotic dosage to preserve sperm motility while realizing the postbiotics’ vaginal health benefits. Further research is warranted to understand the underlying mechanisms and refine practical applications in reproductive health.

## 1. Introduction

A healthy women’s vagina is colonized with various aerobic and anaerobic bacteria, collectively known as the vaginal microbiome [1,2]. Within the vaginas of women of reproductive age more than ten different species can be found. This microbiome has an established role in retaining the physiological function of the female reproductive system, pathogen defense, and preventing urogenital diseases [3,4,5,6,7]. *Lactobacillus* dominates the healthy vaginal microbiota, exerting beneficial effects through various potential mechanisms [8,9,10,11]. Conversely, a lack of dominant *Lactobacillus* is associated with various reproductive system disorders and adverse pregnancy outcomes [12,13], which has led to the concept of vaginal probiotic supplementation as a potential treatment strategy. Therefore, *Lactobacillus*, as the most documented strain used as a probiotic or postbiotic supplement, has been suggested as an adjuvant to antibiotics to mitigate the potential antimicrobial resistance [14,15,16], especially in the treatment of various obstetric and gynecological conditions [17]. Even though probiotics have been proven generally safe, people are still concerned about the potential risk of administering “live bacteria” in some specific situations, such as pregnancy or active preparation for motherhood pregnancy [18,19]. On the other hand, maintaining the stability of probiotic products during the rigorous handling, transportation, and storage to ensure the presence of a sufficient number of viable bacteria (probiotics) to confer the benefits of probiotics brings about some practical challenges in the industrial production and marketing phase [20]. Due to these challenges, postbiotics, inactivated probiotics, and products formed when probiotic bacteria break down have attracted a great deal of research focus, resulting in growing evidence on the ability to promote health and treat disease through modulation of the gut microbiota [21].

In 2021, ISAPP (The International Scientific Association of Probiotics and Prebiotics) issued a postbiotic consensus statement, defining postbiotics as a preparation of inanimate microorganism and/or their components (including just the fermentation supernatant and/or bacterial cell wall and content) that are helpful to the host’s health [20]. Therefore, postbiotics research is gaining widespread interest in modern medicine, with compelling evidence supporting their efficacy as therapeutic and preventive agents, exhibiting anti-inflammatory, immunomodulatory, antioxidant, and anti-cancer properties [22].

The potential benefits of *Lactobacillus*-based bioactive compounds (postbiotics) on vaginal health have also gained increasing research attention in recent years [23,24,25,26]. A recent study demonstrated the positive impact of *Lactobacillus*-based postbiotics, administered in the form of vaginal gel, on BV [27]. While probiotic and postbiotic interventions can potentially enhance the female host’s ability to reproduce, the sperm would encounter a significantly higher concentration of *Lactobacillus* and *Lactobacillus*-based postbiotics in the vagina. Post-liquefaction, unprotected sperm navigate the vaginal environment to cross the cervix. At this stage, the liquid *Lactobacillus*-rich vaginal environment including both vaginal and probiotic secretions plays a delicate role in sperm viability and function [28,29], consequently affecting fertilization and pregnancy rates.

Despite the availability of several vaginal probiotic suppositories as commercial over-the-counter supplements (e.g. Ecovag^®^, Deerland Probiotics & Enzymes A/S, Hundested, Denmark), the potential impact and dose dependency of the resulting increase in the concentration of postbiotics on human sperm quality has not yet been thoroughly investigated.

In the context of a post-ejaculation in vivo scenario, in addition to postbiotics, sperm quality is influenced by both the beneficial and detrimental effects of live and active vaginal microbiota [30] and the inherent dynamics of the female reproductive system [31]. Additionally, probiotics may induce different effects under different bacterial concentrations [32]. The varying composition of the microbiota and, consequently, postbiotics in different individuals could also affect the results and make them incomparable.

Conventional semen analysis, notably concentration, motility, and morphology, remains the standard for evaluating male fertility potential [33]. While routine semen analyses are imperative in the initial identification and evaluation of the severity of male factor infertility [33,34], their limited scope does not allow them to fully elucidate sperm fertilization potential or identify the underlying etiology of infertility, especially in cases of unexplained infertility [35]. Several studies have emphasized the correlation between sperm DNA damage and its impact on fertilization and embryo development [36,37,38]. Clinical studies have also shown that the DNA fragmentation index assessed by the sperm chromatin dispersion (SCD) test [39,40] alongside routine semen analysis provides a much more accurate diagnosis of IVF outcomes [41].

Therefore, to rigorously evaluate the potential effects of *Lactobacillus rhamnosus* PB01 (DSM 14870) postbiotics on sperm quality, this study was designed as a controlled in vitro experiment. By conducting the study in vitro, we aimed to eliminate potential confounding factors from the female reproductive tract and physical effects from vaginal bacteria, such as the reduction in sperm motility due to physical adherence [30]. In addition to routine semen analysis, sperm DNA fragmentation index (DFI), and morphology were assessed to provide a comprehensive evaluation of sperm quality. To the best of our knowledge, this is the first prospective controlled in vitro study to assess the effects of a postbiotic on motility, kinematic parameters, DNA fragmentation, and morphology of normozoospermic human sperm.

## 2. Materials and Methods

### 2.1. Ethical Approval

This study was performed at the Department of Health Science and Technology at Aalborg University (Aalborg, Denmark), following approval from the Scientific Ethics Committee of the Northern Jutland Region, Denmark, according to the committee law (§ 14, stk. 1, cf. § 2, nr. 1–3). Prior to the experiment, all participants received written and oral information about the study, provided signed informed consent, and completed a comprehensive health questionnaire.

### 2.2. Study Population

Healthy male participants aged between 18 to 30 were included in this study. Subjects exhibiting ejaculatory disorders or impaired semen quality due to any known genetic abnormalities like Y-microdeletions and abnormal karyotypes were not included. History of diabetes, cardiovascular disease, vasectomy, orchitis, removed one testicle, ejaculatory disorders, and psychological illnesses requiring continuous medical treatment, use of antibiotics, cyclosporine, allopurinol, glucocorticoids, colchicine, or antidepressants within the last three months were also considered as exclusion factors.

### 2.3. Preparation of Postbiotics 

The postbiotics were prepared by incubating 1 × 10^14^ CFUs of *L. rhamnosus* PB01 (ADM Denmark A/S, Hundested, Denmark) in 15 mL “De Man, Rogosa, and Sharpe” (MRS) broth (Thermo Fisher, Waltham, MA, USA) at 37 °C for 45 h under anaerobic conditions. The supernatant was collected following centrifugation of the suspension at 1000× *g* for 10 min at 4 °C. Any residual bacteria were removed by filtering the supernatant through a 0.22 μm Millipore filter aliquoted in 5 mL batches and stored at −18 °C until use. Before the experiment, the pH of the thawed (at room temperature) supernatant was adjusted to neutral (pH = 7) using 1 M NaOH and used to prepare the different postbiotic concentrations.

### 2.4. Semen Collection and Experimental Protocols

Participants were asked to maintain 3–7 days of sexual abstinence before semen samples were collected by masturbation into a sterile cup in a private room close to the laboratory. The samples were weighted to evaluate volume and allowed to liquefy for 30–45 min at room temperature. After liquefaction, 10 µL of the fresh semen was loaded onto a 20 µm deep chamber of a Leja slide (Leja, Nieuw-Vennep, The Netherlands) to assess sperm concentration and motility by the Sperm Class Analyzer (SCA^®^; version 5.6; Microptic S.L., Barcelona, Spain) computer-aided sperm analysis (CASA) system, ensuring compliance with the inclusion criteria. Samples with a concentration ≥15 million/mL, total sperm count ≥39 million/sample, total motility ≥40%, and progressive motility ≥32% were considered normozoospermic (according to the WHO 2010 criteria [42]) and included in this study. Each of the included samples was divided into seven parts (200 μL each) and mixed with 200 μL Pure Sperm Wash (SW; Nidacon, Gothenburg, Sweden) as control, required concentrations of MRS Broth in SW to form 50% (MRS50), 15% (MRS15), and 5% (MRS5) sham groups, and different concentrations of postbiotics from *L. rhamnosus* (PB01) in SW to form the 50% (PB50), 15% (PB15), and 5% (PB5) postbiotic groups.

#### 2.4.1. Motility and Kinematic Parameters

Sperm motility (including the percentage of total motile sperm, progressive sperm, non-progressive sperm, and immotile sperm), detailed kinematic parameters, and percentage of hyperactivated sperm were evaluated by the Motility/Concentration module of the SCA^®^ CASA system at baseline (T1), and after 60 min (T2) and 90 min (T3) of incubation at room temperature.

In brief, samples from each group were loaded into a 20 µm deep chamber slide. One-second videos from at least five different fields (or more to ensure a minimum of 400 sperm) along the center of the chamber were captured (50 fps) using a Basler Scout A780–54fc camera (Basler, Ahrensburg, Germany), mounted on a Nikon Eclipse 50i microscope with a 10× positive phase contrast objective (Nikon, Minato city, Tokyo, Japan) and a green filter, as previously described by Dardmeh et al. [43,44]. The SCA^®^ software was used to analyze the recorded videos, while an expert technician manually corrected any possible errors in the detected spermatozoa. The SCA^®^ categorized spermatozoa into progressively motile (STR > 80%), non-progressively motile (80% > STR > 0%), and immotile (STR = 0%) according to WHO criteria [42]. The different sperm categories and kinematic parameters assessed by the Sperm Class Analyzer^®^ computer-aided sperm analysis system are presented in Table 1. A representative example of the sperm trajectory detected by the SCA^®^ CASA system is presented in Supplementary File S1.

The assessed kinematic parameters can be divided into velocity parameters, including straight-line velocity (VSL), curvilinear velocity (VCL), average path velocity (VAP), and motion-path parameters, including linearity (LIN), straightness (STR), wobble (WOB), the amplitude of lateral head displacement (ALH), and beat-cross-frequency (BCF), as previously described by Alipour et al. [45]. Hyperactivated sperm motility was classified as 150 < VCL (μm/s) < 500, Lin (%) < 50%, and ALH (μm) > 3.5 [45].

#### 2.4.2. Sperm DNA Fragmentation Index (DFI)

DFI assessment was evaluated based on the Sperm Chromatin Dispersion (SCD) test [46] using the GoldCyto Sperm DNA kit (Goldcyto Biotech Corp., Guangzhou, China) according to the manufacturer’s instructions. Due to the limited volume of samples, evaluation of DFI at baseline and after 90 min of incubation at room temperature in different groups was performed in two subsets of the samples. Five randomly selected samples were used to evaluate DFI in the control and high-concentration sham (MRS50) and postbiotic (PB50) groups (subset A), while another five randomly selected samples were used to evaluate DFI in the Control, and PB50, PB15, and PB5 test groups (Subset B).

For each sample, A minimum of 400 spermatozoa were observed using the 20× objective (brightfield), and the halo sizes were quantified and evaluated by the DNA module of the SCA^®^. Spermatozoa demonstrating a halo width equivalent to or less than one-third of the core’s diameter and those with no halo were considered “spermatozoa with DNA fragmentation” and degraded, respectively. The DFI was calculated as the percentage of fragmented and degraded spermatozoa to the total number of cells counted.

#### 2.4.3. Morphology

The percentage of sperm with normal morphology was evaluated in the subsets described above at baseline and after 90 min of incubation at room temperature.

In brief, a routine sperm smear was fixed in SpermBlue^®^ fixative (Microptic, Barcelona, Spain) for 10 min, stained using the SpermBlue^®^ stain (Microptic, Barcelona, Spain) for 8 min, washed by gently dipping in distilled water, and air-dried at room temperature. The slides were then evaluated using a 60× objective (brightfield) and the SCA^®^ morphology module, which automatically quantified sperm head and midpiece measurements [47]. An expert technician controlled all assessments and corrected possible errors.

### 2.5. Statistical Analysis

Data were analyzed for normal distribution using the Shapiro–Wilk test. Data are presented as means ± standard deviation (SD) unless stated otherwise. A repeated measures analysis of variance (ANOVA) was used to compare the motility, kinematic parameters, hyperactive motility, and morphology among the groups and at different time points. Pair-wise comparisons were performed using the Bonferroni post hoc test, wherever ANOVA yielded a statistically significant difference. A simple effects analysis was used to compare the percentage of mucus penetration ability and DFI values among groups at different time points. All statistical analyses were performed using the SPSS software (Version 29; IBM Corporation, Armonk, NY, USA). *p* < 0.05 was considered significant.

## 3. Results

A preliminary investigation revealed that the spermatozoa exhibited complete immobility when exposed to postbiotic solutions higher than 50%. Consequently, postbiotic concentrations of PB5, PB15, and PB50 were included in this study.

This study included 18 healthy male participants with a mean age of 25.37 (±3.39) years. All 18 samples were examined for sperm motility, kinematic parameters, hyperactive sperm. Five randomly selected samples were evaluated for the percentage of normal morphology and sperm DNA fragmentation index.

### 3.1. Seminal Parameters in Donor Samples

Table 2 presents the seminal characteristics, motility categorization (according to WHO 5 criteria [42]), and kinematic details for samples from the participating donors.

### 3.2. The Effect of Different Postbiotic Doses on Sperm Motility

The higher concentration postbiotic (PB50) group demonstrated significantly lower percentages of total motile sperm (*p* < 0.01) compared to the control (SW), higher concentration sham (MRS50), medium (PB15), and lower concentration (PB5) of postbiotic groups throughout the study. However, in medium (PB15) and lower concentrations of the postbiotic groups (PB5), no difference in motile sperm was observed at any time compared with the control and sham groups.

**Figure 1:**
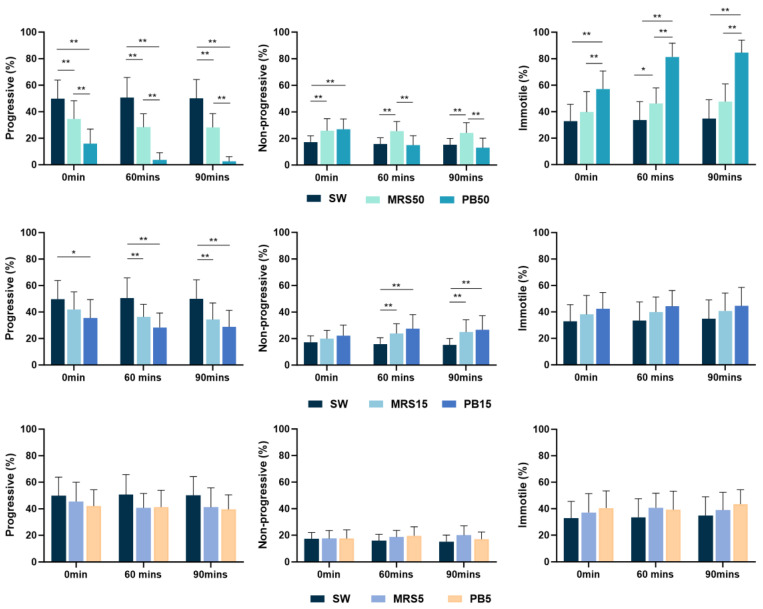
Mean percentage of progressively motile, non-progressively motile, and immotile spermatozoa, incubated in control (SW), 5% MRS broth (MRS5), 15% MRS broth (MRS15), 50% MRS broth (MRS50), 5% postbiotics (PB5), 15% postbiotics (PB15), and 50% postbiotics (PB50) at baseline (0 min), and after 60 and 90 min of incubation (n = 18). Error bars demonstrate standard deviation. * marks *p* < 0.05, ** marks *p* < 0.01.

The higher concentration postbiotic solution (PB50) showed significantly lower (*p* < 0.01) progressive motility compared to the control group (SW) and sham group (MRS50) at all time points. The percentage of progressively motile spermatozoa in the sham (MRS50) group demonstrated significantly lower values than the control group (SW) at all time points (Figure 1).

The PB15 solution indicated significantly lower progressive motility (*p* < 0.05) compared with the control group throughout the study. In contrast, the lower concentration postbiotic (PB5) showed no significant difference compared to the control group throughout the study (Figure 1).

**Figure 2:**
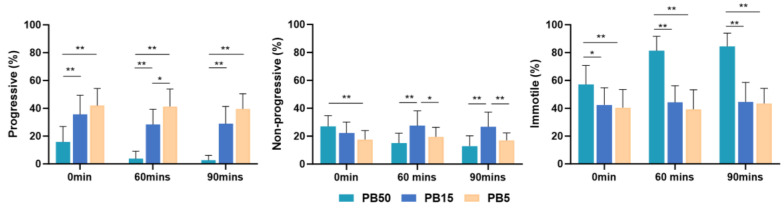
Mean percentage of progressively motile, non-progressively motile, and immotile spermatozoa, incubated in 5% postbiotics (PB5), 15% postbiotics (PB15), and 50% postbiotics (PB50) at baseline (0 min), and after 60 and 90 min of incubation (n = 18). Error bars demonstrate standard deviation. * marks *p* < 0.05, ** marks *p* < 0.01.

The percentage of progressive sperm in the higher concentration postbiotic (PB50) group was significantly lower (*p* < 0.01) than in the medium and lower concentration of the postbiotic solutions (PB15 and PB5) group at all time points. Progressive sperm in the PB15 group showed a significantly lower value (*p* < 0.01) than the lower concentration of the postbiotic solutions (PB5) at 60 min of incubation (Figure 2).

In higher concentrations (PB50) of the postbiotic solution, the percentage of progressive sperm decreased over time. However, there was no difference in the lower postbiotic group (PB5) concentration throughout the study.

Both the PB50 and MRS50 demonstrated higher non-progressive sperm motility immediately after incubation (*p* < 0.05) compared with the control group (SW). However, lower postbiotic (PB5) concentration did not exhibit a significant difference at any time compared with the control group (Figure 1). Notably, non-progressive motility in the higher-concentration postbiotic (PB50) group was significantly higher (*p* < 0.05) than in the lower-concentration postbiotic (PB5) group (Figure 2).

The higher concentration postbiotic (PB50) group demonstrated higher percentages of immotile sperm (*p* < 0.01) compared to the control (SW) (Figure 1), medium (PB15) (*p* < 0.05), and low postbiotic group (PB5) (*p* < 0.01) throughout the study (Figure 2).

In both the PB50 and sham (MRS50) groups, the immotile sperm value was significantly higher in 60 min compared to the baseline. The rest of the groups did not show changes over time.

The percentage of hyperactivated sperm showed no significant difference between groups throughout the study.

### 3.3. The Effect of Different Postbiotic Doses on Kinematic Parameters

**Figure 3:**
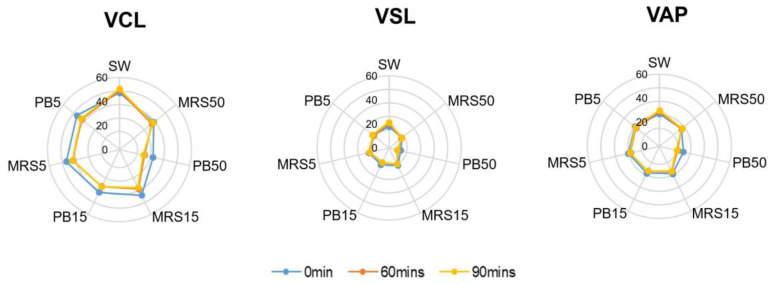
Velocity parameters in control (SW), 5% MRS broth (MRS5), 15% MRS broth (MRS15), 50% MRS broth (MRS50), 5% postbiotics (PB5), 15% postbiotics (PB15), and 50% postbiotics (PB50) at baseline (0 min), and after 60 and 90 min of incubation (n = 18). The radius demonstrates the percentage of sperm in the respective group. VCL: Curvilinear velocity; VSL: Straight-line velocity; VAP: Average path velocity.

The control group contained higher mean velocity values of VCL, VSL, and VAP compared with the higher postbiotic group (PB50) (*p* < 0.01) at all time points. Lower concentration of the postbiotic group (PB5) showed no difference compared with the control group at baseline but lower values of VCL, VSL, and VAP after 60 min (*p* < 0.01) of incubation (Figure 3).

**Figure 4:**
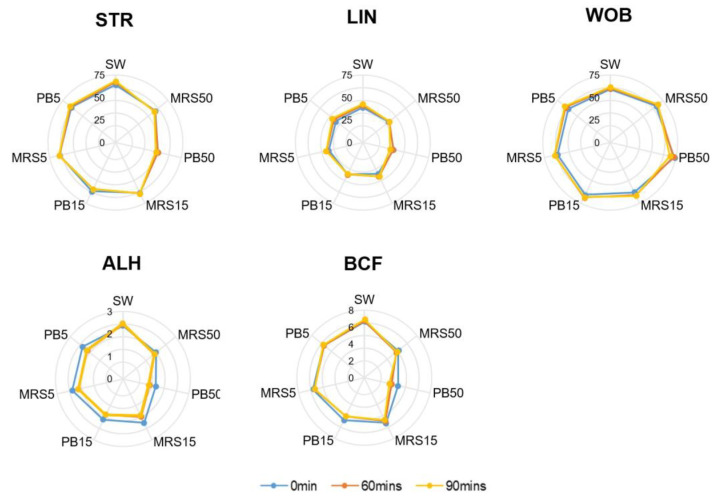
Motion-path parameters in control (SW), 5% MRS broth (MRS5), 15% MRS broth (MRS15), 50% MRS broth (MRS50), 5% postbiotics (PB5), 15% postbiotics (PB15), and 50% postbiotics (PB50) at baseline (0 min), and after 60 and 90 min of incubation (n = 18). The radius demonstrates the percentage of sperm in the respective group. Lin: Linearity; STR: Straightness; WOB: Wobble, ALH: Amplitude of lateral head displacement; BCF: Beat cross frequency (BCF).

The mean motion-path parameters of STR, WOB, and BCF were significantly higher (*p* < 0.05) in the control group (SW) compared with the PB50 group at all time points. Still, they presented no significant difference between the PB5) and the control group at any time. The mean ALH in the PB5 group showed no difference from the control at baseline but a significantly lower value after 60 min of incubation. No difference in LIN was detected among all groups at all time points (Figure 4).

### 3.4. The Effect of Different Postbiotic Doses on Sperm DNA Fragmentation Index

**Figure 5:**
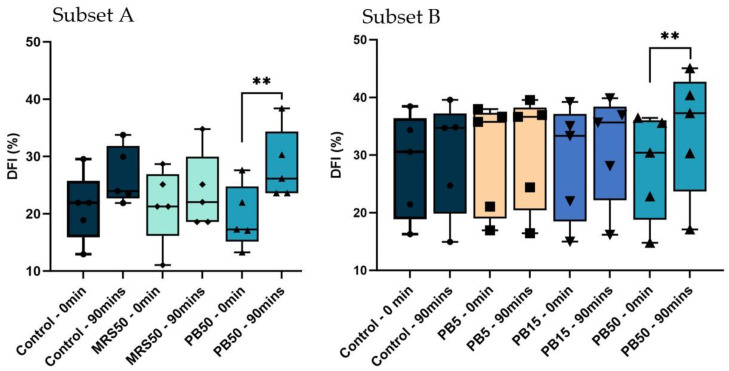
Box and whisker plots demonstrating the percentage of sperm DNA fragmentation index. (DFI) in control (SW), 5% postbiotics (PB5), 15% postbiotics (PB15), and 50% postbiotics (PB50) at baseline (0 min), and after 90 min of incubation (n = 5) in sample subsets A (n = 5), and B (n = 5). The box spans the interquartile range (25–75); geometric symbols (squares, triangles, and circles) denote individual data points; whiskers demonstrate the range (min, max), and the horizontal line inside the box presents the median; ** *p* < 0.01.

The PB50 group exhibited a significantly higher DFI after 90 min of incubation compared with baseline (*p* < 0.01) in both subsets of samples. The control and MRS50 in subset A, and control, PB15, and PB5 in subset B did not significantly differ over time. Furthermore, there were no significant differences among any groups in any subsets at baseline or after 90 min of incubation (Figure 5).

### 3.5. The Effect of Different Postbiotic Doses on Sperm Morphology

The percentage of sperm with normal morphology did not show differences between any of the groups compared to baseline and 90 min of incubation in the two subsets. Furthermore, there was no difference between the three postbiotic and control groups at baseline and 90 min of incubation.

## 4. Discussion

This manuscript presents the results of the first prospective in vitro study investigating the impact of postbiotics on sperm motility, comprehensive kinematic parameters morphology, and DNA fragmentation in normozoospermic men. The findings underscore the safety of a PB5 of *Lactobacillus rhamnosus* PB01 (DSM-14870) postbiotics, confirming their benign effect on sperm quality.

The indirect effects of the probiotics have been associated with the bioactive compounds secreted by them or released after their disintegration and lysis, also called “postbiotics” [21], which can contain cell components such as lipopolysaccharide, peptidoglycan, lipoteichoic acid, extracellular vesicles and enzymes, peptides, organic acids, and other bioactive compounds [48]. A recent clinical trial demonstrated the efficacy of postbiotics from *Lactobacillus paracasei* ProSci-92 and *Lactobacillus rhamnosus* ProSci-109 in alleviating the clinical symptoms associated with bacterial vaginitis indicated by improvement of abnormal vaginal secretions, through increasing the relative abundance of vaginal *Lactobacillus* and regulating the vaginal microbiota composition [27]. The postbiotic in the mentioned study was prepared as a gel by incorporating the fermentation solution from 1 × 10^6^ *L. paracasei* ProSci-92 and 1 × 10^6^ *L. rhamnosus* ProSci-109 (fermented to pH 4.60 at 37 °C) with adding Kabo 940, triethanolamine, propylene glycol, PEG-90M, moisturizing gel, and phenoxyethanol), and a daily dose of 3 mg was administered into the deep part of the vagina using a gel catheter for one week [27]. A healthy vagina generally produces 4.65 ± 1.8 mg of vaginal discharge in 24 h [49]; thus, the 3 mg gel would have resulted in a vaginal postbiotic concentration between 48.72% to 68.25%. In such a scenario, the elevated level of postbiotics could also exert effects on spermatozoa deposited within the vaginal environment.

The current study assessed the potential effect of *L. rhamnosus* PB01 fermentation solution (culture supernatant) on sperm quality in vitro. This solution would contain non-viable bacterial-free extracts and bioactive compounds secreted by the probiotics or released after their disintegration and lysis, which would be considered *L. rhamnosus* postbiotics according to the definition. Postbiotics are considered superior to probiotics owing to their higher safety, and economic benefits while providing the same beneficial therapeutic effects as probiotics [22]. They have the advantage of ease of use and storage, stability in a broad range of environments, and simultaneously deliver beneficial effects in metabolic, immunomodulatory, anti-cancer, and antioxidant functions [50,51].

Our initial pilot study (results not presented) demonstrated that a postbiotic concentration higher than 50% resulted in complete loss of motility in all spermatozoa within 30 min in vitro. Therefore, only the PB15 and PB5 postbiotics were considered as test groups, while the PB50 was kept in the study as the negative control group.

Sperm motility is a key predictor of male fertility [52]. The percentage of total motile sperm did not differ between the control, PB5, and PB15 groups. However, further categorizing the sperm as progressively motile and non-progressively motile sperm [53] could provide additional biological insight and serve as a diagnostic and prognostic factor, particularly with pregnancy rates [54,55]. The results showed a significant drop in the percentage of progressively motile sperm in the PB15 compared to the control group throughout the study. In contrast, PB5 did not show any difference. Thus, it is safe to speculate that PB15 negatively affected the successful conception rate.

Around 30–45 min after ejaculation, the semen sample liquefies, and the spermatozoa reach their peak motility, allowing the sperm to leave the seminal plasma and enter the cervical mucus. The spermatozoa that remain in the vagina will start losing their motility within the next 30 min, with the majority gradually becoming immotile within 120 min [56]. Thus, the presented study assessed the progressive motility of sperm immediately after exposure, as well as at 60- and 90-minute post-exposure to postbiotics.

The kinematic parameters reflected these trends, with PB50 showing significantly lower VSL, VCL, VAP, STR, WOB, ALH, and BCF values at all time points. While PB5 did not show any effect at baseline, VCL, VSL, and VAP demonstrated lower amounts after 60 min of incubation. These changes were, however, not substantial enough to affect the percentage of total motile and progressive spermatozoa in PB5, implying that the lower concentrations of postbiotic supplementation would not affect the sperm’s ability to reach the oocyte during migration within the female reproductive tract.

Although the specific mechanisms underlying postbiotic bioactivities remain unclear and may vary across different target hosts, there are indications that postbiotics share mechanisms of action similar to probiotics in certain cases [20]. In line with this assumption, the significantly lower progressive sperm motility in the PB50 group complies with the previous findings on the potential inhibitory effects of probiotics [30,32,57].

The study by Li et al. [58] illustrated that *Lactobacillus* can adhere and adversely affect intracellular Ca^2+^ concentrations in sperm. Ca^2+^ plays a pivotal role in regulating sperm motility and successful fertilization [59,60]. Thus, it can be suggested that vaginal postbiotic suppositories might impact sperm function and motility by blocking the intake and consequently reducing the intracellular concentrations of essential nutrients (e.g., Ca^2+^). Furthermore, probiotics could also indirectly affect the sperm through their secretions. Fujita et al. elucidated that lipopolysaccharide (LPS) located on the bacterial surface possesses a detrimental capacity by selectively interacting with Toll-like receptor 2 (TLR2) situated on the acrosome surface, resulting in diminished motility of human sperm and the initiation of spermatozoa apoptosis [61,62]. On the other hand, probiotic secretions could also provide a more favorable environment for the spermatozoa by impeding the proliferation of pathogenic microorganisms through the secretion of diverse secondary metabolites with antibacterial properties (e.g., lactic acid, hydrogen peroxide, and biosurfactants [63]). These findings can be considered as potential mechanisms of action of postbiotics.

Several studies have demonstrated the positive regulatory impact of oral probiotic consumption on sperm quality via the modulation of the reproductive hormone levels or anti-inflammatory response in male animal models [64,65] and humans [66,67]. Concerning postbiotics, a study by Díaz et al. [68] examined the impact of oral postbiotics on sperm motility. This study, conducted on rabbits, revealed that 11 weeks of oral supplementation with postbiotics based on lactic acid bacteria led to fewer abnormal spermatozoa and increased acrosome integrity. Another study on rats reported that intraperitoneal injection of *L. rhamnosus* GR-1 supernatant helped reduce inflammation caused by lipopolysaccharide and the incidence of preterm birth in pregnant mice [69].

However, it is important to note that oral administration of probiotics and postbiotics, as well as intraperitoneal administration of postbiotics, will result in a systemic effect, while the vaginal microbiome, probiotics, or postbiotics delivered via intravaginal suppository could also have a more local impact, potentially affecting the sperm deposited in the vagina through their byproducts [61,62,63], physically adhering to them [30,32,58], or indirectly via their effect on the vaginal environment [27,70].

Spermatozoa do not possess a DNA damage repair system [71]. Consequently, excessive oxidative stress or other substances in seminal plasma (e.g., free nuclease) can disrupt sperm DNA integrity post-ejaculation [72,73], potentially negatively affecting fertilization rate and embryo development [74,75].

The PB50 exhibited a significantly higher DFI, while the PB15 and PB5 showed no change in DFI after 90 min of incubation. This suggests that lower doses of postbiotics did not affect sperm DNA quality or perhaps even played a protective role against the negative effect of incubation time on DNA integrity [76].

Previous studies have shown that oral probiotic supplementation can reduce DFI in human sperm [77,78]. However, there is a gap in the literature regarding the effect of postbiotics on sperm DNA integrity. Our findings offer an initial perspective on the in vitro effect of postbiotics on sperm DFI, but further comprehensive studies are required to establish the in vivo and in vitro effects of postbiotics on sperm DFI.

Sperm structure and morphology predominantly develop during spermatogenesis [79]. However, certain environmental factors may affect some morphological parameters, as evidenced by the hypo-osmotic swelling test [80]. Despite this, morphology assessments showed no difference in any groups at any time during the study, suggesting that post-ejaculation exposure to postbiotics did not adversely affect sperm morphology.

However, it is important to recognize certain limitations in our study. Firstly, the study focused on particular levels of *L. rhamnosus* postbiotics and is not indicative of postbiotics from the diverse spectrum of probiotics in the reproductive tract [81]. Additionally, this study was carried out in vitro and not equipped to assess the underlying mechanisms of action or the potential effect of the probiotic secretions in the physiologically dynamic environment of the vagina, in the presence of the vaginal cells and their secretions, or through their effects on pathogenic microorganisms in the vaginal area. Thus, further in vitro and in vivo studies are required to validate these findings and evaluate the potential effects of the probiotic secretions on sperm quality, fertilization rates, and pregnancy outcomes in sub-par samples, unfavorable conditions, or in the presence of pathogenic microorganisms.

## 5. Conclusions

The results of this study showed that lower concentrations (5%) of *Lactobacillus rhamnosus* PB01 (DSM 14870) postbiotic did not adversely impact sperm quality in normozoospermic samples in vitro. However, 15% postbiotic concentration significantly reduced the percentage of progressively motile sperm, while a 50% concentration resulted in a significant increase in the percentage of immotile sperm immediately after exposure, with this effect continuing to increase up to 90 min. Sperm morphology was not affected by postbiotic supplementation. However, 90 min of exposure to 50% concentration resulted in a significant increase in sperm DNA fragmentation. This underscores the necessity of further studies to strike a balance between the advantageous vaginal effects and preserving optimal sperm functional quality, during the production and utilization of suppositories, particularly in couples attempting conception. Subsequent research is warranted to elucidate potential mechanisms, establish a “safe dose” of *Lactobacillus* concentration, and refine practical recommendations for applications in reproductive health.

## Figures and Tables

**Table 1:** The different sperm motility categories according to WHO 5 criteria [42], and kinematic parameters assessed by the Sperm Class Analyzer^®^ computer-aided sperm analysis system (adapted from Alipour et al. 2017 [45]).

Parameter/Unit	Description of the Parameter
**Motility categories**	
PR (%)	Spermatozoa move actively, linearly or in a large circle, regardless of speed
NP (%)	All other patterns of motility with an absence of progression
IM (%)	No movement
**Kinematic parameters**	
VCL (μm/s)	Curvilinear velocity along the actual swimming path
VSL (μm/s)	Straight-line velocity along shortest path from start to end point
VAP (μm/s)	Average path velocity based on every 11th frame of VCL path
LIN (%)	Linearity of a curvilinear path expressed as VSL/VCL
STR (%)	Straightness expressed as VSL/VAP
WOB (%)	Oscillation index expressed as VSL/VAP
ALH (μm)	Amplitude of lateral head displacement
BCF (Hz)	Beat cross-frequency based on VCL crossing VAP per second
Hyperactivated (%)	150 < VCL (μm/s) < 500; Lin (%) < 50%; ALH (μm) > 3.5 *

PR: Progressively motile spermatozoa; NP: Non-progressively motile spermatozoa; IM: Immotile spermatozoa; VCL: Curvilinear velocity; VAP: Average path velocity; VSL: Straight line velocity; STR: Straightness; LIN: Linearity index; WOB: Wobble; ALH: Amplitude of lateral head displacement; BCF: Best cross frequency. * Most CASA systems use ALH Max, which is ~2 × ALH.

**Table 2:** Seminal characteristics and kinematic details of donor samples (*n* = 18).

Parameter	Median (25–75 Percentiles)
**Seminal characteristics**	
Volume (mL)	3.47 (2.57–5.05)
Concentration (M/mL)	50.8 (32.1–62.31)
Motile sperm (%)	f80.72 (62.25–89.23)
**Motility categorization** (**WHO 5 criteria**)	
PR %	58.63 (40.62–66.68)
NP %	20.39 (18.17–26.1)
IM %	19.28 (10.77–37.75)
**Kinematic details**	
VCL	40.38 (34.36–46.55)
VAP	25.22 (21.74–28.57)
VSL	15.86 (14.99–19.48)
STR	63.43 (60.32–66.45)
LIN	40.65 (35.74–43.13)
WOB	62.66 (58.88–63.68)
ALH	1.97 (1.82–2.33)
BCF	6.18 (5.51–6.86)

PR: Progressively motile spermatozoa; NP: Non-progressively motile spermatozoa; IM: Immotile spermatozoa; VCL: Curvilinear velocity; VAP: Average path velocity; VSL: Straight line velocity; STR: Straightness; LIN: Linearity index; WOB: Wobble; ALH: Amplitude of lateral head displacement; BCF: Best cross frequency.

## Data Availability

The data supporting this study’s findings are available from the corresponding author, [FD], upon reasonable request.

## Supplementary Materials

The following supporting information can be downloaded at: https://www.mdpi.com/article/10.3390/nu16111781/s1, Figure S1: A representative example of sperm motility tracks in different experimental groups.

## References

[1] Petrova M.I., Lievens E., Malik S., Imholz N., Lebeer S. (2015). Lactobacillus Species as Biomarkers and Agents That Can Promote Various Aspects of Vaginal Health. Front. Physiol..

[2] Ravel J., Gajer P., Abdo Z., Schneider G.M., Koenig S.S.K., McCulle S.L., Karlebach S., Gorle R., Russell J., Tacket C.O. (2011). Vaginal Microbiome of Reproductive-Age Women. Proc. Natl. Acad. Sci. USA.

[3] Gajer P., Brotman R.M., Bai G., Sakamoto J., Schütte U.M.E., Zhong X., Koenig S.S.K., Fu L., Ma Z., Zhou X. (2012). Temporal Dynamics of the Human Vaginal Microbiota. Sci. Transl. Med..

[4] Barrientos-Durán A., Fuentes-López A., de Salazar A., Plaza-Díaz J., García F. (2020). Reviewing the Composition of Vaginal Microbiota: Inclusion of Nutrition and Probiotic Factors in the Maintenance of Eubiosis. Nutrients.

[5] Donders G.G.G., Bosmans E., Dekeersmaeckerb A., Vereecken A., Van Bulck B., Spitz B. (2000). Pathogenesis of Abnormal Vaginal Bacterial Flora. Am. J. Obs. Gynecol..

[6] Wiesenfeld H.C., Hillier S.L., Krohn M.A., Landers D.V., Sweet R.L. (2003). Bacterial Vaginosis Is a Strong Predictor of *Neisseria gonorrhoeae* and *Chlamydia trachomatis* Infection. Clin. Infect. Dis..

[7] Younes J.A., Lievens E., Hummelen R., van der Westen R., Reid G., Petrova M.I. (2018). Women and Their Microbes: The Unexpected Friendship. Trends Microbiol..

[8] Zhang Z., Lv J., Pan L., Zhang Y. (2018). Roles and Applications of Probiotic Lactobacillus Strains. Appl. Microbiol. Biotechnol..

[9] Valenti P., Rosa L., Capobianco D., Lepanto M.S., Schiavi E., Cutone A., Paesano R., Mastromarino P. (2018). Role of Lactobacilli and Lactoferrin in the Mucosal Cervicovaginal Defense. Front. Immunol..

[10] Aroutcheva A., Gariti D., Simon M., Shott S., Faro J., Simoes J.A., Gurguis A., Faro S. (2001). Defense Factors of Vaginal Lactobacilli. Am. J. Obs. Gynecol..

[11] Delgado-Diaz D.J., Jesaveluk B., Hayward J.A., Tyssen D., Alisoltani A., Potgieter M., Bell L., Ross E., Iranzadeh A., Allali I. (2022). Lactic Acid from Vaginal Microbiota Enhances Cervicovaginal Epithelial Barrier Integrity by Promoting Tight Junction Protein Expression. Microbiome.

[12] Tsai H., Tsui K., Chiu Y., Wang L. (2023). Adverse Effect of Lactobacilli-depauperate Cervicovaginal Microbiota on Pregnancy Outcomes in Women Undergoing Frozen–Thawed Embryo Transfer. Reprod. Med. Biol..

[13] Fettweis J.M., Serrano M.G., Brooks J.P., Edwards D.J., Girerd P.H., Parikh H.I., Huang B., Arodz T.J., Edupuganti L., Glascock A.L. (2019). The Vaginal Microbiome and Preterm Birth. Nat. Med..

[14] Aslam B., Khurshid M., Arshad M.I., Muzammil S., Rasool M., Yasmeen N., Shah T., Chaudhry T.H., Rasool M.H., Shahid A. (2021). Antibiotic Resistance: One Health One World Outlook. Front. Cell Infect. Microbiol..

[15] Nataraj B.H., Mallappa R.H. (2021). Antibiotic Resistance Crisis: An Update on Antagonistic Interactions between Probiotics and Methicillin-Resistant Staphylococcus Aureus (MRSA). Curr. Microbiol..

[16] Elshaghabee F.M.F., Rokana N. (2022). Mitigation of Antibiotic Resistance Using Probiotics, Prebiotics and Synbiotics. A Review. Env. Chem. Lett..

[17] Kesmodel U.S., Dardmeh F., Alipour H. (2021). Probiotics in Obstetrics and Gynecology—Where Is the Future?. Acta Obs. Gynecol. Scand..

[18] Zhang T., Zhang W., Feng C., Kwok L.-Y., He Q., Sun Z. (2022). Stronger Gut Microbiome Modulatory Effects by Postbiotics than Probiotics in a Mouse Colitis Model. NPJ Sci. Food.

[19] Suez J., Zmora N., Segal E., Elinav E. (2019). The Pros, Cons, and Many Unknowns of Probiotics. Nat. Med..

[20] Salminen S., Collado M.C., Endo A., Hill C., Lebeer S., Quigley E.M.M., Sanders M.E., Shamir R., Swann J.R., Szajewska H. (2021). The International Scientific Association of Probiotics and Prebiotics (ISAPP) Consensus Statement on the Definition and Scope of Postbiotics. Nat. Rev. Gastroenterol. Hepatol..

[21] Nataraj B.H., Ali S.A., Behare P.V., Yadav H. (2020). Postbiotics-Parabiotics: The New Horizons in Microbial Biotherapy and Functional Foods. Microb. Cell Fact..

[22] Żółkiewicz J., Marzec A., Ruszczyński M., Feleszko W. (2020). Postbiotics—A Step Beyond Pre- and Probiotics. Nutrients.

[23] Tachedjian G., Aldunate M., Bradshaw C.S., Cone R.A. (2017). The Role of Lactic Acid Production by Probiotic Lactobacillus Species in Vaginal Health. Res. Microbiol..

[24] Abbasi A., Aghebati-Maleki L., Homayouni-Rad A. (2022). The Promising Biological Role of Postbiotics Derived from Probiotic *Lactobacillus* Species in Reproductive Health. Crit. Rev. Food Sci. Nutr..

[25] Abramov V., Khlebnikov V., Kosarev I., Bairamova G., Vasilenko R., Suzina N., Machulin A., Sakulin V., Kulikova N., Vasilenko N. (2014). Probiotic Properties of Lactobacillus Crispatus 2029: Homeostatic Interaction with Cervicovaginal Epithelial Cells and Antagonistic Activity to Genitourinary Pathogens. Probiotics Antimicrob. Proteins.

[26] Croatti V., Parolin C., Giordani B., Foschi C., Fedi S., Vitali B. (2022). Lactobacilli Extracellular Vesicles: Potential Postbiotics to Support the Vaginal Microbiota Homeostasis. Microb. Cell Fact..

[27] Shen X., Xu L., Zhang Z., Yang Y., Li P., Ma T., Guo S., Kwok L.-Y., Sun Z. (2023). Postbiotic Gel Relieves Clinical Symptoms of Bacterial Vaginitis by Regulating the Vaginal Microbiota. Front. Cell Infect. Microbiol..

[28] Romero R., Hassan S.S., Gajer P., Tarca A.L., Fadrosh D.W., Nikita L., Galuppi M., Lamont R.F., Chaemsaithong P., Miranda J. (2014). The Composition and Stability of the Vaginal Microbiota of Normal Pregnant Women Is Different from That of Non-Pregnant Women. Microbiome.

[29] Sellami H., Znazen A., Sellami A., Mnif H., Louati N., Ben Zarrouk S., Keskes L., Rebai T., Gdoura R., Hammami A. (2014). Molecular Detection of Chlamydia Trachomatis and Other Sexually Transmitted Bacteria in Semen of Male Partners of Infertile Couples in Tunisia: The Effect on Semen Parameters and Spermatozoa Apoptosis Markers. PLoS ONE.

[30] Wang H., Chen T., Chen Y., Luo T., Tan B., Chen H., Xin H. (2019). Evaluation of the Inhibitory Effects of Vaginal Microorganisms on Sperm Motility in Vitro. Exp. Ther. Med..

[31] Suarez S.S., Pacey A.A. (2006). Sperm Transport in the Female Reproductive Tract. Hum. Reprod. Update.

[32] Zhang F., Dai J., Chen T. (2021). Role of Lactobacillus in Female Infertility Via Modulating Sperm Agglutination and Immobilization. Front. Cell Infect. Microbiol..

[33] Barbăroșie C., Agarwal A., Henkel R. (2021). Diagnostic Value of Advanced Semen Analysis in Evaluation of Male Infertility. Andrologia.

[34] Agarwal A., Bui A.D. (2017). Oxidation-Reduction Potential as a New Marker for Oxidative Stress: Correlation to Male Infertility. Investig. Clin. Urol..

[35] Hamada A., Esteves S.C., Agarwal A. (2011). Unexplained Male Infertility. Hum. Androl..

[36] Rasmussen J.M.K., Dalgaard M.I.R., Alipour H., Dardmeh F., Christiansen O.B. (2024). Seminal Oxidative Stress and Sperm DNA Fragmentation in Men from Couples with Infertility or Unexplained Recurrent Pregnancy Loss. J. Clin. Med..

[37] Zheng W.-W., Song G., Wang Q.-L., Liu S.-W., Zhu X.-L., Deng S.-M., Zhong A., Tan Y.-M., Tan Y. (2018). Sperm DNA Damage Has a Negative Effect on Early Embryonic Development Following in Vitro Fertilization. Asian J. Androl..

[38] Lewis S.E.M., Aitken R.J. (2005). DNA Damage to Spermatozoa Has Impacts on Fertilization and Pregnancy. Cell Tissue Res..

[39] Lu J.C., Jing J., Chen L., Ge Y.F., Feng R.X., Liang Y.J., Yao B. (2018). Analysis of Human Sperm DNA Fragmentation Index (DFI) Related Factors: A Report of 1010 Subfertile Men in China. Reprod. Biol. Endocrinol..

[40] Yang H., Li G., Jin H., Guo Y., Sun Y. (2019). The Effect of Sperm DNA Fragmentation Index on Assisted Reproductive Technology Outcomes and Its Relationship with Semen Parameters and Lifestyle. Transl. Androl. Urol..

[41] Okubo T., Onda N., Hayashi T., Kobayashi T., Omi K., Segawa T. (2023). Performing a Sperm DNA Fragmentation Test in Addition to Semen Examination Based on the WHO Criteria Can Be a More Accurate Diagnosis of IVF Outcomes. BMC Urol..

[42] World Health Organization (2010). WHO Laboratory Manual for the Examination and Processing of Human Semen.

[43] Dardmeh F., Alipour H., Nielsen H.I., Rasmussen S., Gazerani P. (2017). Effects of Chronic Musculoskeletal Pain on Fertility Potential in Lean and Overweight Male Patients. Pain. Res. Manag..

[44] Dardmeh F., Heidari M., Alipour H. (2021). Comparison of Commercially Available Chamber Slides for Computer-Aided Analysis of Human Sperm. Syst. Biol. Reprod. Med..

[45] Alipour H., Van Der Horst G., Christiansen O.B., Dardmeh F., Jørgensen N., Nielsen H.I., Hnida C. (2017). Improved Sperm Kinematics in Semen Samples Collected after 2 h versus 4–7 Days of Ejaculation Abstinence. Hum. Reprod..

[46] Fernández J.L., Muriel L., Goyanes V., Segrelles E., Gosálvez J., Enciso M., LaFromboise M., De Jonge C. (2005). Simple Determination of Human Sperm DNA Fragmentation with an Improved Sperm Chromatin Dispersion Test. Fertil. Steril..

[47] Van Der Horst G., Maree L. (2010). SpermBlue®: A New Universal Stain for Human and Animal Sperm Which Is Also Amenable to Automated Sperm Morphology Analysis. Biotech. Histochem..

[48] Cuevas-González P.F., Liceaga A.M., Aguilar-Toalá J.E. (2020). Postbiotics and Paraprobiotics: From Concepts to Applications. Food Res. Int..

[49] Godley M.J. (1985). Quantitation of Vaginal Discharge in Healthy Volunteers. BJOG.

[50] Bourebaba Y., Marycz K., Mularczyk M., Bourebaba L. (2022). Postbiotics as Potential New Therapeutic Agents for Metabolic Disorders Management. Biomed. Pharmacother..

[51] Aguilar-Toalá J.E., Garcia-Varela R., Garcia H.S., Mata-Haro V., González-Córdova A.F., Vallejo-Cordoba B., Hernández-Mendoza A. (2018). Postbiotics: An Evolving Term within the Functional Foods Field. Trends Food Sci. Technol..

[52] Fernández-López P., Garriga J., Casas I., Yeste M., Bartumeus F. (2022). Predicting Fertility from Sperm Motility Landscapes. Commun. Biol..

[53] World Health Organization (2021). WHO Laboratory Manual for the Examination and Processing of Human Semen.

[54] Simon L., Lewis S.E.M. (2011). Sperm DNA Damage or Progressive Motility: Which One Is the Better Predictor of Fertilization in Vitro?. Syst. Biol. Reprod. Med..

[55] Vogiatzi P., Pouliakis A., Sakellariou M., Athanasiou A., Athanasiou A., Colaghis A., Finelli R., Loutradis D., Henkel R., Agarwal A. (2022). Male Age and Progressive Sperm Motility Are Critical Factors Affecting Embryological and Clinical Outcomes in Oocyte Donor ICSI Cycles. Reprod. Sci..

[56] Brannigan R.E., Lipshultz L.I. (2008). Sperm Transport and Capacitation. Glob. Libr. Women’s Med..

[57] Answal M., Prabha V. (2018). Escherichia Coli Recombinant Sperm Immobilizing Factor RecX as a Potential Vaginal Contraceptive. Reprod. Biol. Endocrinol..

[58] Li P., Wei K., He X., Zhang L., Liu Z., Wei J., Chen X., Wei H., Chen T. (2021). Vaginal Probiotic Lactobacillus Crispatus Seems to Inhibit Sperm Activity and Subsequently Reduces Pregnancies in Rat. Front. Cell Dev. Biol..

[59] Finkelstein M., Etkovitz N., Breitbart H. (2020). Ca^2+^ Signaling in Mammalian Spermatozoa. Mol. Cell Endocrinol..

[60] Quill T.A., Sugden S.A., Rossi K.L., Doolittle L.K., Hammer R.E., Garbers D.L. (2003). Hyperactivated Sperm Motility Driven by CatSper2 Is Required for Fertilization. Proc. Natl. Acad. Sci. USA.

[61] Fujita Y., Mihara T., Okazaki T., Shitanaka M., Kushino R., Ikeda C., Negishi H., Liu Z., Richards J.S., Shimada M. (2011). Toll-like Receptors (TLR) 2 and 4 on Human Sperm Recognize Bacterial Endotoxins and Mediate Apoptosis. Hum. Reprod..

[62] Chapot-Chartier M.-P., Kulakauskas S. (2014). Cell Wall Structure and Function in Lactic Acid Bacteria. Microb. Cell Fact..

[63] Borges S., Silva J., Teixeira P. (2014). The Role of Lactobacilli and Probiotics in Maintaining Vaginal Health. Arch. Gynecol. Obs..

[64] Dardmeh F., Alipour H., Gazerani P., van der Horst G., Brandsborg E., Nielsen H.I. (2017). Lactobacillus Rhamnosus PB01 (DSM 14870) Supplementation Affects Markers of Sperm Kinematic Parameters in a Diet-Induced Obesity Mice Model. PLoS ONE.

[65] Rahimiyan-Heravan M., Roshangar L., Karimi P., Sefidgari-Abrasi S., Morshedi M., Saghafi-Asl M., Bavafa-Valenlia K. (2020). The Potential Therapeutic Effects of Lactobacillus Plantarum and Inulin on Serum and Testicular Reproductive Markers in Diabetic Male Rats. Diabetol. Metab. Syndr..

[66] Helli B., Kavianpour M., Ghaedi E., Dadfar M., Haghighian H.K. (2022). Probiotic Effects on Sperm Parameters, Oxidative Stress Index, Inflammatory Factors and Sex Hormones in Infertile Men. Hum. Fertil..

[67] Akram M., Ali S.A., Kaul G. (2023). Probiotic and Prebiotic Supplementation Ameliorates Chronic Restraint Stress-Induced Male Reproductive Dysfunction. Food Funct..

[68] Díaz Cano J.V., Argente M.-J., García M.-L. (2021). Effect of Postbiotic Based on Lactic Acid Bacteria on Semen Quality and Health of Male Rabbits. Animals.

[69] Yang S., Li W., Challis J.R.G., Reid G., Kim S.O., Bocking A.D. (2014). Probiotic Lactobacillus Rhamnosus GR-1 Supernatant Prevents Lipopolysaccharide-Induced Preterm Birth and Reduces Inflammation in Pregnant CD-1 Mice. Am. J. Obs. Gynecol..

[70] Hanson L., VandeVusse L., Jermé M., Abad C.L., Safdar N. (2016). Probiotics for Treatment and Prevention of Urogenital Infections in Women: A Systematic Review. J. Midwifery Womens Health.

[71] González-Marín C., Gosálvez J., Roy R. (2012). Types, Causes, Detection and Repair of DNA Fragmentation in Animal and Human Sperm Cells. Int. J. Mol. Sci..

[72] Har-Vardi I., Mali R., Breietman M., Sonin Y., Albotiano S., Levitas E., Potashnik G., Priel E. (2007). DNA Topoisomerases I and II in Human Mature Sperm Cells: Characterization and Unique Properties. Hum. Reprod..

[73] Tvrdá E., Arroyo F., Gosálvez J. (2018). Dynamic Assessment of Human Sperm DNA Damage I: The Effect of Seminal Plasma-Sperm Co-Incubation after Ejaculation. Int. Urol. Nephrol..

[74] Bungum M. (2012). Sperm DNA Integrity Assessment: A New Tool in Diagnosis and Treatment of Fertility. Obs. Gynecol. Int..

[75] Ribas-Maynou J., Novo S., Torres M., Salas-Huetos A., Rovira S., Antich M., Yeste M. (2022). Sperm DNA Integrity Does Play a Crucial Role for Embryo Development after ICSI, Notably When Good-Quality Oocytes from Young Donors Are Used. Biol. Res..

[76] Sabbaghian M., Hosseinifar H., Rafaee A., Gilani M.S. (2022). Assessment of the Impact Induced by Different Incubation Time, Storage Time, Storage Medium and Thawing Methods on Sperm DNA Fragmentation Assay: A before–after Study. J. Hum. Reprod. Sci..

[77] Corbett G., Crosby D., McAuliffe F. (2021). Probiotic Therapy in Couples with Infertility: A Systematic Review. Eur. J. Obstet. Gynecol. Reprod. Biol..

[78] Valcarce D.G., Genovés S., Riesco M.F., Martorell P., Herráez M.P., Ramón D., Robles V. (2017). Probiotic Administration Improves Sperm Quality in Asthenozoospermic Human Donors. Benef. Microbes.

[79] Sharma R., Agarwal A. (2011). Spermatogenesis: An Overview. Sperm Chromatin.

[80] Ramu S., Jeyendran R.S. (2013). The Hypo-Osmotic Swelling Test for Evaluation of Sperm Membrane Integrity. Spermatogenesis Methods Protoc..

[81] Feng T., Liu Y. (2022). Microorganisms in the Reproductive System and Probiotic’s Regulatory Effects on Reproductive Health. Comput. Struct. Biotechnol. J..

