# Supplementary material for: Effect of Postbiotics Derived from Lactobacillus rhamnosus PB01 (DSM 14870) on Sperm Quality: A Prospective In Vitro Study
Source: Nutrients. 2024 Jun 6;16(11):1781. doi: 10.3390/nu16111781 (PMC11174611; doi:10.3390/nu16111781)
Supplement: Supplementary file 1 [file nutrients-16-01781-s001.zip › nutrients-3024525-supplementary.pdf]

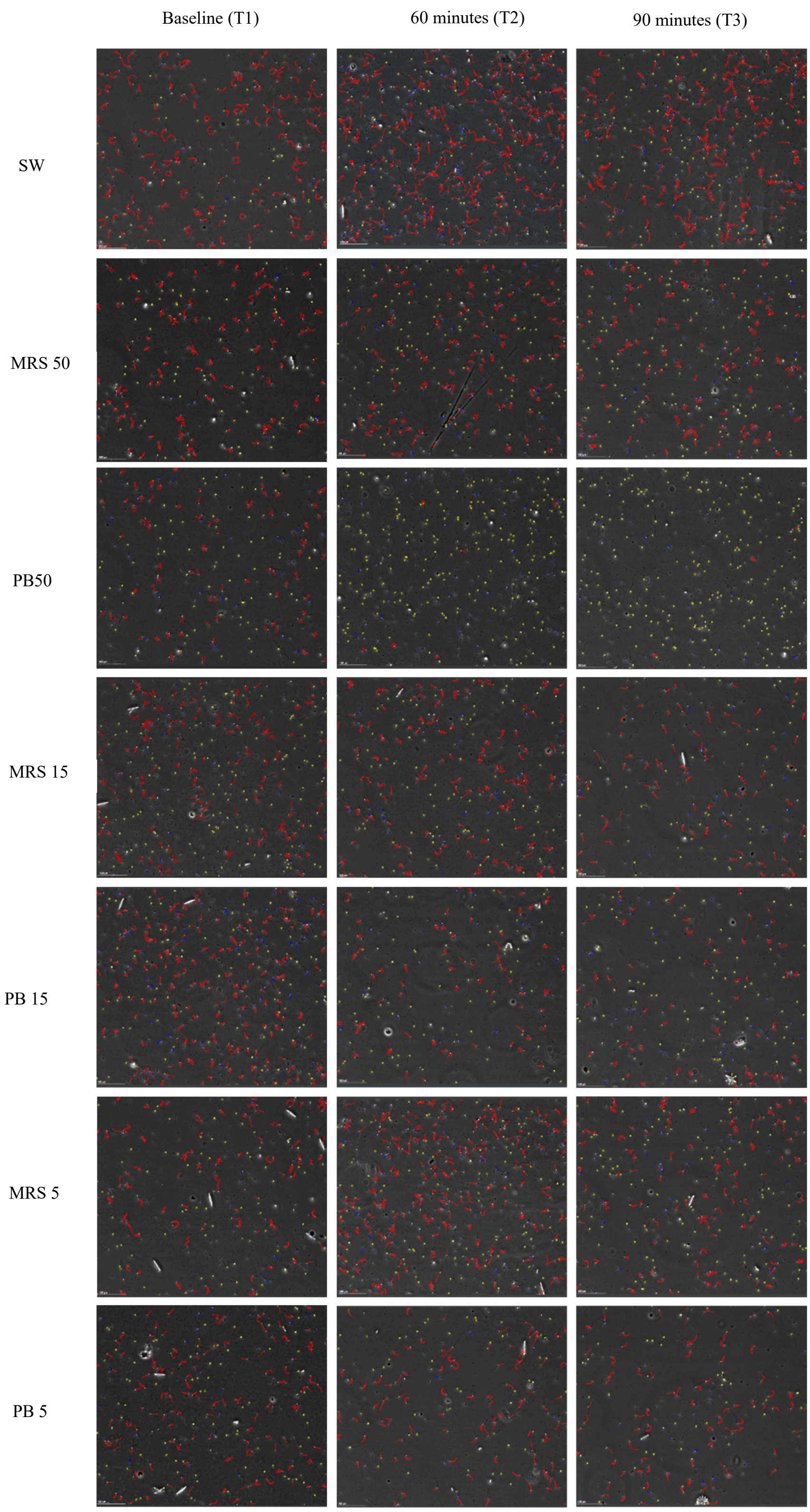

Figure S1. A representative example of sperm motility tracks in different experimental groups captured by computer-assistant sperm analysis (CASA) system (magnification, x100) at baseline (T1, 0 minutes), and after 60 mins (T2), and 90 minutes(T3) of incubation in G1: Pure Sperm Wash (SW), G2: 50% concentration of MRS broth (MRS50), G3: 50% concentration of postbiotics (PB50), G4: 15% concentration of MRS broth (MRS15), G5: 15% concentration of postbiotics (PB15), G6: 5% concentration of MRS broth (MRS5) and G7: 5% concentration of postbiotics (PB5) ; Scale bar: 100 µm. Red tracks represent progressive sperm. Blue tracks represent non-progressive sperm. Yellow circles represent immotile sperm.
